# Feasibility study on the application of HD-sEMG-based force estimation technology in the assessment of hand dysfunction in cerebral palsy

**DOI:** 10.3389/fbioe.2025.1580098

**Published:** 2025-04-02

**Authors:** Xinlu Zhang, Kun Wang, De Wu, Xu Zhang, Xiang Chen

**Affiliations:** ^1^ School of Microelectronics, University of Science and Technology of China, Hefei, Anhui, China; ^2^ Department of Pediatrics, The First Affiliated Hospital of Anhui Medical University, Hefei, Anhui, China

**Keywords:** cerebral palsy, force estimation model, LSTM, sEMG, transfer learning

## Abstract

**Introduction:**

In response to the demand for a quantifiable means for assessing hand dysfunction in cerebral palsy (CP), this paper proposed and conducted a novel high-density (HD)-surface electromyography (sEMG)-based muscle force estimation framework.

**Methods and Results:**

A highly generalized source network was developed firstly based on long short-term memory (LSTM) networks and three different healthy adult HD-sEMG-force datasets, achieving a root mean square error (RMSE) of 6.31% in force estimation across various force modes; Then, transfer learning techniques were applied to fine tune the well-trained source network using data from healthy children, establishing five gesture-specific target networks that achieved RMSE below 10% in force estimation tasks independent of the subjects; Finally, a muscle force estimation experiment was conducted on 16 children with CP using the gesture-specific target networks.

**Conclusion:**

By comparing and analyzing the experimental results of CP group and healthy control group, CP children with different grades of Manual Ability Classification System (MACS), and CP children with different types of symptoms, it was verified that the abnormal EMG-force relationship obtained using the proposed muscle force estimation scheme had the potential for clinical application in the assessment of CP hand dysfunction. Muscle force estimation based on sEMG has broad application prospects in clinical practice. The research work in this paper has important value in promoting the clinical application of muscle force estimation technology based on sEMG, which is conducive to improving the quantitative assessment level of motor dysfunction.

## 1 Introduction

Cerebral palsy (CP) refers to a non-progressive brain injury syndrome caused by various pathogenic factors from fetal to infancy and is a common and serious disabling disease in pediatrics (Bax et al., 2005). The symptoms of cerebral palsy mainly include abnormal muscle tone, limb deformities, motor dysfunction and so on. The mainstream treatment options include rehabilitation training, surgical procedures, and medication relief, among which rehabilitation treatment can help improve motor dysfunction in children with CP in the long term. In rehabilitation treatment, assessing the motor dysfunction of children with CP can help doctors provide more targeted rehabilitation treatments for patients. At present, doctors mainly rely on various assessment scales, such as the Gross Motor Function Measure (GMFM), to assess the motor function of children with CP (Russell et al., 2013). The assessment results largely rely on the clinical experience and subjective judgments of rehabilitation doctors and are sometimes not very accurate. Therefore, there is an urgent need for objective tools to guide the rehabilitation training of CP children. Specifically, quantitative muscle force estimation technology has important clinical application value for assessing CP motor dysfunction.

Muscle force estimation refers to the evaluation of muscle contraction strength, applied in fields such as rehabilitation engineering (Lee et al., 2018), clinical medicine (Mathieu et al., 2023), and human‒computer interaction (Su et al., 2021; Liu et al., 2024). The methods for estimating muscle force can be divided into direct measurement methods and indirect measurement methods. Direct measurement methods obtain muscle strength by placing sensors at the tendon with high accuracy, but their invasiveness limits their application range (Amarantini et al., 2012). Indirect measurement methods, represented by the surface electromyography (sEMG)-based muscle force estimation, are convenient and non-invasive, and so have become an important research direction. sEMG collected by attaching electrodes to the surface of the skin, carries rich information on muscle contraction intensity and timing. sEMG-based force estimation technology has broad application potential in the fields of clinical medicine and rehabilitation engineering. Specifically, because of its ability to detect the functional condition of muscles, it can provide a basis for surgical assessment in clinical medicine (Medved et al., 2020) and is beneficial for customizing personalized rehabilitation plans during the rehabilitation training process (Meattini et al., 2020). Traditional sEMG signals are measured via discrete electrodes, which are easy to attach but are susceptible to cross interference from adjacent muscles and can only provide information on local positions. In contrast, high-density electromyographic signals (HD-sEMG) collected by high-density array electrodes can provide comprehensive muscle activity information and capture complex motion patterns. In recent years, HD-sEMG-based muscle force estimation has attracted considerable research interest.

To establish a nonlinear relationship between sEMG and muscle force effectively, various models have been introduced. Hill model proposed by A. V. Hill in 1938 is the most widely used model. Hill model is a representative physiological model that uses parameters such as muscle fiber contraction speed, muscle fiber length changes, and activation signal intensity during human muscle contraction to model and estimate muscle force (Hill, 1997). Owing to its high complexity, Xiao and Higginson (2010), Ackland et al. (2012), and Bujalski et al. (2018) have been dedicated to simplifying Hill model for practical application (Xiao and Higginson, 2010; Ackland et al., 2012; Bujalski et al., 2018). Various regression models also have been applied to fit the relationship between sEMG and muscle force. AI Harrach et al. (2017) reported through comparative research that among many regression models, third-order or higher-order polynomial models have better performance in force estimation (Al Harrach et al., 2017). In recent years, neural networks have been widely welcomed by researchers in HD-sEMG-based muscle force estimation because of their strong generalizability. Choi et al. (2010) established a fully connected neural network consisting of 12 hidden layer units, achieving good performance in subject-related palm grip estimation, but the model performance was poor in subject-independent scenarios (Choi et al., 2010). Xu et al. (2018) introduced convolutional neural network (CNN), long and short-term memory (LSTM) network, and their combined network structure (C-LSTM) into static isometric elbow flexion tasks (Xu et al., 2018). Xue and Lai. (2023) proposed a one-dimensional convolutional deep neural prediction network and applied it to human‒machine interaction systems to achieve precise control of sEMG-based robots (Xue and Lai, 2023).

With the development of sEMG-based muscle force estimation technology, some researchers are attempting to introduce it into clinical rehabilitation applications (Shan et al., 2025a; Shan et al., 2025b). Mokri C et al. utilized machine learning techniques such as support vector machine (SVM), support vector regression (SVR), and random forest (RF), as well as genetic learning algorithms, to achieve high-precision estimation of lower limb muscle force, with an estimation root mean square error (RMSE) of 6.05%. In addition, they designed a lower limb rehabilitation robot to assist patients in completing knee joint rehabilitation training, greatly improving the effectiveness of rehabilitation treatment (Mokri et al., 2022). Leung et al. applied muscle force estimation techniques to evaluate swallowing in patients with post radiation dysphagia. They generated dynamic topographic maps on the basis of the root mean square (RMS) of HD-sEMG signals to illustrate the function of the anterior cervical muscle during swallowing and evaluated the symmetry of muscle average force and swallowing patterns through objective parameters, including the average RMS, left/right energy ratio and left/right energy difference (Leung et al., 2023). To understand the joint torque generated by the muscles of CP patients during walking and to assist with suboptimal gait patterns, Suncheol Kwon et al. estimated the internal torque of the knee joint using sEMG signals and the knee joint angle. Based on different assumptions, they proposed four estimation models. The results showed that the best estimation model could be selected based on the degree of contraction, with a normalized root mean square error (NRMSE) between 0.15 and 0.29 (Kwon et al., 2012). In summary, the application of sEMG-based muscle force estimation technology in CP rehabilitation is still limited and requires further exploration.

According to the potential need for assessing motor disorders, we have summarized two main application modes of sEMG-based force estimation technology in the clinical assessment of children with CP. One is to predict the muscle force generated during rehabilitation training, and the other is to assess CP motor dysfunction based on differences in the sEMG-force relationship with healthy children of the same age group. When we directly apply the sEMG-based muscle force estimation schemes proposed in existing studies to clinical practice, the following problems may be faced. First, the effectiveness of most schemes has been validated only in fixed force mode, such as the rectangular mode (Hajian et al., 2021; Sittiruk et al., 2025), triangular mode (Mao et al., 2023), and sine mode (Na and Kim, 2017; Simon et al., 2024), and with limited accuracy in arbitrary force modes. However, it is difficult for children with CP to exert force according to a fixed mode. Second, in most earlier studies, the force estimation model was usually established in subject-specific mode, namely, the training data and testing data came from the same subject. However, this method of establishing a specific force estimation model for individuals does not help reveal the abnormal sEMG-force relationship related to individual motor disorders. Therefore, the prerequisite for applying sEMG-based force estimation technology to clinical CP motor dysfunction assessment is to establish a force estimation model that is independent of users and highly generalized to force mode.

With the assessment of hand dysfunction for children with CP from the perspective of an abnormal sEMG-force relationship as the research object, this paper proposes a novel HD-sEMG-based muscle force estimation framework for clinical application. As we know, to establish a force estimation model that is independent of users and highly generalized to force modes, sufficient data covering sEMG‒force relationship across diverse force modes during human movement are needed. However, due to the fact that existing studies on muscle force estimation have focused mostly on healthy adults, the available sEMG-force datasets also come from healthy adults. The novelty of the proposed framework is that it first attempts to establish a highly generalized source network using healthy adult (HA) data covering different force modes; then, it attempts to obtain gesture-specific target networks using gesture action data from healthy children (HC) and transfer learning techniques; and finally, it aims to carry out a clinical hand dysfunction assessment of children with cerebral palsy (CP) using the gesture-specific target networks. As an innovative study that applies HD-sEMG-based muscle force estimation technology to clinical practice, the results of this study are of great significance for promoting the development of quantitative assessment techniques for motor disorders in children with CP.

## 2 Materials and methods

**FIGURE 1:**
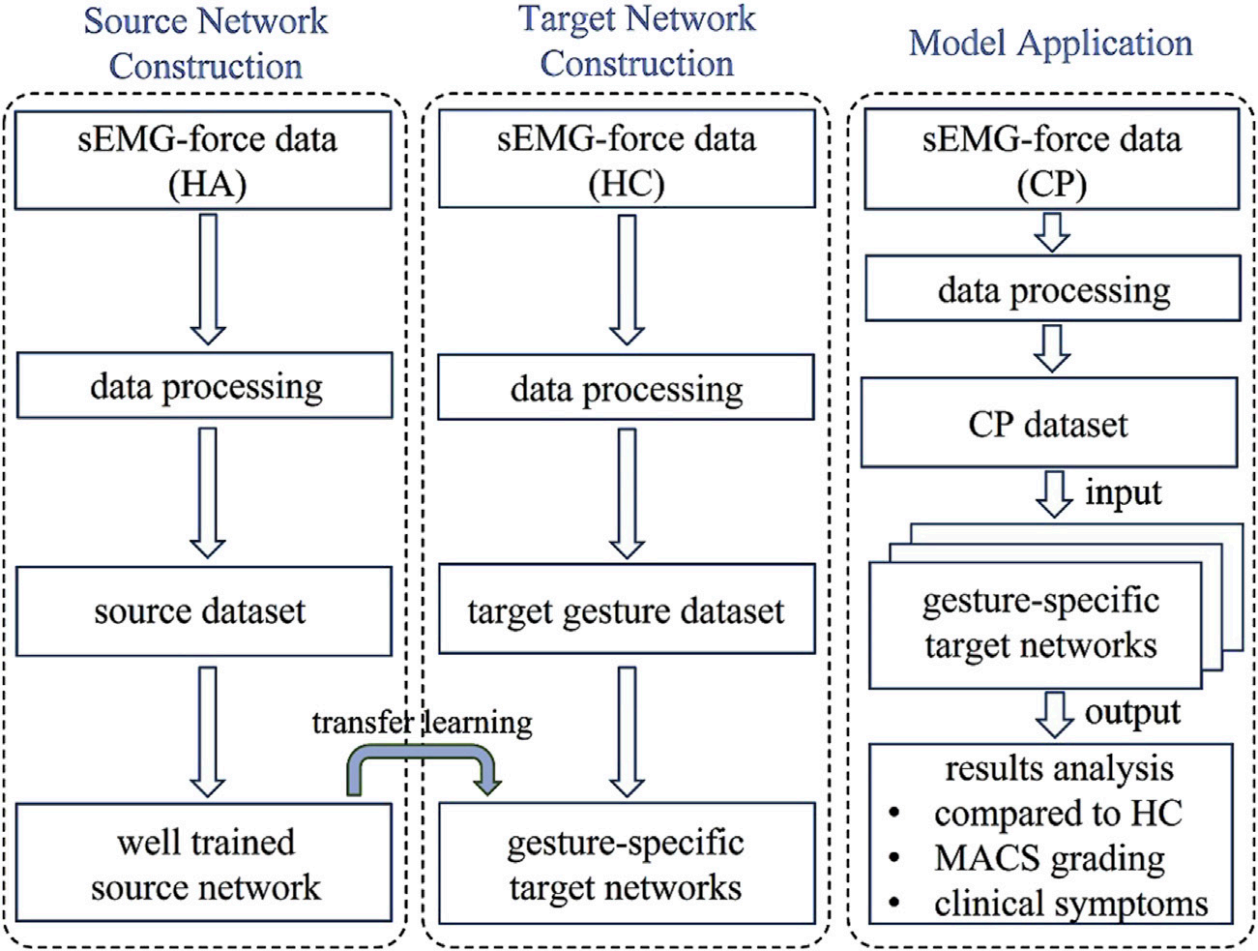
Research route.

This study received approval from the Ethics Review Committee of University of Science and Technology of China under Application No. 2022-N(H)-150. The proposed HD-sEMG-based muscle force estimation framework is shown in Figure 1 and includes three parts: source network construction, target networks construction and model application. To construct the source network, a force estimation model was established on the basis of a LSTM network, and three healthy adult (HA) HD-sEMG-force datasets with different force modes were used as the source dataset to train it to obtain a well-trained source network. To construct the target networks, five target gestures were first defined according to the needs of hand function rehabilitation in children with CP, and then HD-sEMG-force data of the target gestures were collected from healthy children (HC) to calibrate the well-trained source network to obtain the gesture-specific target networks. Specifically, this study adopted transfer learning techniques on the well-trained source network and established five gesture-specific target networks named M1-M5 tailored to corresponding target gestures. In the model application part, with the goal of providing clinical rehabilitation physicians with more objective and quantitative assessment criteria, thereby facilitating improved rehabilitation treatment, using the five gesture-specific muscle force estimation models M1-M5, the differences in the sEMG‒force relationship between CP subjects and healthy children of the same age group were analyzed firstly, then the application value of sEMG-based muscle force estimation in the hand dysfunction assessment of children with CP was explored from the perspectives of Manual Ability Classification System (MACS) grading and clinical symptom manifestations.

### 2.1 Datasets

#### 2.1.1 Source dataset

The three HD-sEMG-force datasets, namely, Dataset A (Zhang et al., 2019), Dataset B (Xu et al., 2018), and Dataset C (Hu et al., 2022), were established by the author’s research team using self-developed equipment, and all consist of 128-channel sEMG signals and the corresponding one-channel force signals. Dataset A comprises the data of the brachial and biceps muscles during isometric contraction elbow flexion tasks from 10 healthy males (aged 24.8 ± 2.6 years, weight 67.2 ± 5.3 kg, and height 174.7 ± 3.9 cm). Specifically, each subject completed static isometric contraction tasks at three levels of force, namely, 20%, 40%, and 60% MVC (Maximum voluntary contraction). The force mode follows an increasing-plateau model (3s for the increasing phase and 3s for the plateau phase). The signal sampling rate is 1 kHz. Dataset B comprises the data of the biceps brachii during isometric contraction elbow flexion task, with the subject performing tasks at three force levels, namely, 35%, 50%, and 65% MVC. The force mode follows an increasing-plateau model (2s for the increasing phase and 4s for the plateau phase). The signal sampling rate is also 1 kHz. Notably, Dataset B includes muscle fatigue data acquired as subjects exerted force until exhaustion. All 24 healthy subjects were recruited and categorized into three groups by age and sex: young males (aged 23.8 ± 2.2 years, height 177.4 ± 5.3 cm, weight 69.5 ± 5.8 kg); young females (aged 22.5 ± 1.4 years, height 161.5 ± 3.6 cm, weight 46.6 ± 5.7 kg); and middle-aged and elderly males (aged 52.5 ± 5.5 years, height 168.3 ± 3.9 cm, weight 71.9 ± 5.7 kg). In this study, data from 11 subjects (3 young males, 3 young females and 5 middle-aged and elderly males) were adopted for source network training. Dataset C comprises the data of forearm flexor under 11 gestures, which are extracted from the common types of gestures in daily life, involving pressure, pinch, grip, and the twist, involving five fingers. Subjects of Dataset C were 10 healthy males aged 23–27 years, and executed three force modes for each gesture: two regular force modes and random force mode. The regular force modes consist of a sine wave force ranging between 0% and 60% MVC (5s) and a constant force at 40% MVC (5s). In the random force mode, the subjects performed each gesture action in a non-fixed mode of their own choice for 20 s. The signal sampling rate is 2 kHz. The samples obtained from datasets A, B and C after processing form the source dataset, with a total of 43,479 sEMG-force samples.

#### 2.1.2 Target gestures and target gesture dataset

**FIGURE 2:**
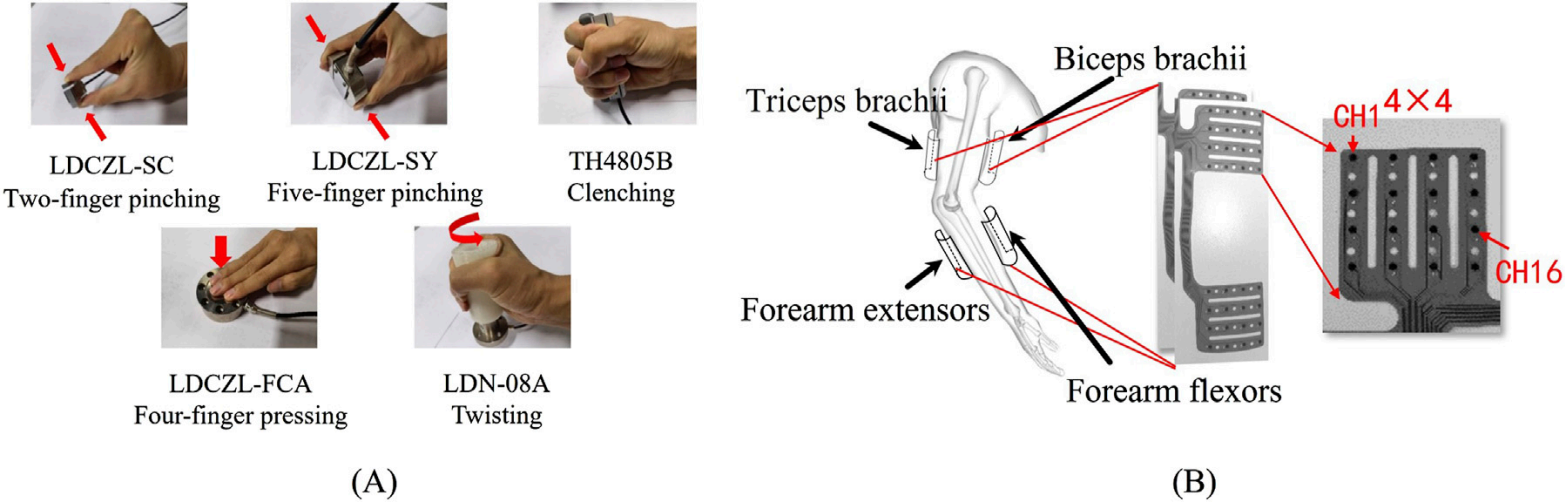
**(A)** Five target gestures and force sensor placement; **(B)** HD-sEMG grids and their placement.

Taking into account the fundamental need for hand function rehabilitation in children with cerebral palsy (CP), this study focused on five gestures involving grasping, pinching, and twisting. Specifically, these gestures encompass two-finger pinching (G1), five-finger pinching (G2), clenching (G3), four-finger pressing (G4), and twisting (G5), as illustrated in Figure 2A.

A total of 16 healthy children (HC), comprising 8 males (aged 7.5 ± 2.0 years) and 8 females (aged 10.3 ± 3.7 years), were recruited from the families of faculty members at the University of Science and Technology of China and the outpatient department of the Pediatric Neurorehabilitation Center at Anhui Medical University. All the HC subjects had no history of neuromuscular injury or related ailments. Guardians of the subjects were briefed on the experimental procedures and signed informed consent forms.

Considering that children have thinner arms, HD-sEMG data were collected using a self-developed 64-channel HD-sEMG grid (size: 4 pieces×4 rows × 4 lines, inter-electrode distance: 14 mm, electrode diameter: 3.5 mm). The electrode grid, which is fabricated from flexible polyimide material, better conforms to the skin. To establish high-precision target networks, it is desirable to collect sEMG from as many parts as possible. Therefore, four electrode grids were positioned on the biceps, triceps, extensor and flexor muscles of the forearm, as depicted in Figure 2B. To accurately obtain the force signal generated during different gesture executions, different force sensors (LOADING SEN, China) were placed as shown in Figure 2B. The LDCZL-SC sensor was used to collect pressure signals from the pinching action of the thumb and index finger, the LDCZL-SY sensor was used to collect pressure signals from the pinching action of the thumb and the other four fingers, the TH4805B sensor was used to collect grip force signals generated by the full palm grip, the LDCZL-FCA sensor was used to collect pressure signals generated by the four fingers pressing down, and the LDN-08A sensor was used to collect torque signals generated by twisting in a fist posture, which could be converted into torsion force through the relationship with the force arm. The sampling rate for each channel of HD-sEMG and the force signal was set to 2 kHz.

The experimental protocol required subjects to uniformly increase their force until 80% MVC and maintain it for 3–5 s during the execution of the five gestures. The force mode was ‘increasing-plateau’. Each subject repeated the force mode eight times for each gesture, with a 15 s break between each repetition. Given the comparatively weaker fine motor control abilities of children than adults, the experimental protocol for collecting HD-sEMG-force data from healthy children focused more on the entire process of applying and releasing force each time, without strict requirements for force mode or maximum force values. The samples obtained from HC data after processing form the target gesture dataset.

#### 2.1.3 CP dataset

A total of 16 children with CP (aged 7.5 ± 3.2 years) were recruited from the Pediatric Neurorehabilitation Center of the First Affiliated Hospital of Anhui Medical University. The recruitment criteria for CP subjects included a clinical diagnosis of congenital cerebral palsy, age between 4 and 16 years, upper limb motor dysfunction attributed to cerebral palsy, no obvious cognitive impairment, and the ability to independently execute target gestures under the guidance of the experimenter. The clinical information of the 16 CP subjects is shown in Table 1. Before the experiment, professional clinical physicians assessed the upper limb motor function of the recruited CP subjects using the MACS grading scale and described their upper limb symptoms in detail. The MACS is mainly used to assess children’s ability to control their hands for functional activities in daily life and is divided into five levels from Ⅰ to Ⅴ, with higher levels indicating poorer hand function (Eliasson et al., 2006). Owing to the loss of control over objects, we could not collect data from CP of grade Ⅴ. In addition, the upper limb symptoms of the 16 CP subjects were categorized into four categories by clinical physicians, namely, Type I to Type IV. Type I was characterized by good hand joint activity, stiffness in upper or forearm muscles, and abnormal muscle tone; Type II had good hand motor function, high gesture discrimination, and no significant stiffness in upper limb muscles; Type III had poor joint mobility in the upper limbs, with symptoms such as finger interlocking, joint stiffness, and wrist drooping; and Type IV had poor ulnar radial separation due to underdeveloped hand function.

**TABLE 1:** Clinical information of 16 CP subjects.

Subject	Gender	Age (year)	Type of CP	MACS	Upper limb symptoms
CP1	F	5.5	right hemiplegia	Ⅱ	Ⅰ
CP2	F	16.0	spastic quadriplegia	Ⅰ	Ⅱ
CP3	M	6.5	spastic quadriplegia	Ⅰ	Ⅱ
CP4	M	10.0	right hemiplegia	Ⅳ	Ⅲ
CP5	M	5.0	right hemiplegia	Ⅳ	Ⅲ
CP6	F	8.0	right hemiplegia	Ⅲ	Ⅰ
CP7	M	7.0	spastic quadriplegia	Ⅱ	Ⅳ
CP8	M	4.0	spastic quadriplegia	Ⅰ	Ⅱ
CP9	M	6.0	right hemiplegia	Ⅰ	Ⅳ
CP10	F	9.0	spastic quadriplegia	Ⅲ	Ⅳ
CP11	M	6.0	spastic quadriplegia	Ⅱ	Ⅰ
CP12	M	9.0	mixed type cerebral palsy	Ⅲ	Ⅲ
CP13	M	8.0	spastic quadriplegia	Ⅱ	Ⅲ
CP14	M	13.0	dyskinetic Cerebral Palsy	Ⅳ	Ⅰ
CP15	M	9.0	right hemiplegia	Ⅰ	Ⅱ
CP16	M	9.3	right hemiplegia	Ⅱ	Ⅱ

The experimental protocol for the data collection of children with CP referred to that of healthy children and was adjusted appropriately according to the CP subject’s status during the experiment. Some CP subjects might have stiff finger joints and required assistance with grasping force sensors (TH4805B). Owing to the basic loss of fine motor function in the hands, CP4 only repeated each gesture four times and had not completed data collection for G4 and G5; CP1-CP3 failed to complete data collection for G3 because of equipment malfunction. All other CP subjects completed the data collection for eight repetitions of each gesture, with each repetition consisting of approximately 10,000 sample points (5 s). In addition, a tracking experiment, in which two data collections with a 6-month interval (named Exp. 1 and Exp. 2) were conducted on CP6 and CP7. CP7 failed to complete data collection for G3 in Exp. 1 because of equipment malfunction. The samples obtained from CP data after processing form the CP dataset.

### 2.2 Data processing

To obtain the standard samples for the input of the force estimation model, some preprocessing steps were performed on HD-sEMG and force signals in source dataset, target gesture dataset and CP dataset.

#### 2.2.1 Normalization and bandpass filtering

Initially, the signal quality of each channel in HD-sEMG was screened, and the defective channels were identified and replaced. Owing to the different sampling rates across the three datasets, bilinear interpolation was subsequently employed to upsample the data in Dataset A and Dataset B. Finally, the HD-sEMG data were filtered via a finite impulse response (FIR) bandpass filter (100th-order FIR filter, Hanning window, 20Hz–500Hz) to eliminate low-frequency noise and extraneous high-frequency irrelevant information.

#### 2.2.2 PCA spatial filtering

PCA can reduce the dimensionality of high-dimensional data through orthogonal decomposition and reconstruction, increasing interpretability while minimizing information loss, and can be used to remove random noise (Staudenmann et al., 2006). We adopted PCA to decompose HD-sEMG into several principal components, which was the same as the number of channels in HD-sEMG, and reconstructed the signal after removing the components with the highest and lowest contribution rates. The reason for this is that we believe the principal component with the highest contribution rate contains redundant common information, whereas the principal component with the lowest contribution rate contains measurement noise. The reconstructed signal then underwent full-wave rectification and low-pass filtering (100th-order FIR filter, Hanning window, cutoff frequency: 5 Hz) to derive HD-sEMG envelope matrix. Finally, the HD-sEMG envelope matrix was normalized with the maximum-minimum method as Formula 1. x represents the envelope sequence of each channel of HD-sEMG, $x_{\min}$ is the minimum value of x , $x_{\max}$ is the maximum value, and *N* is the length.

$${x_{i}}^{\prime }=\frac{x_{i}-x_{\min}}{x_{\max}-x_{\min}}\;i=1,2,\ldots ,N$$ (1)

#### 2.2.3 NMF-based channel optimization

The NMF-based channel optimization method proposed in our previous work (Huang et al., 2017) was used to decompose the HD-sEMG envelope matrix into an activation mode matrix and the corresponding activation coefficient matrix first. Each row of the activation coefficient matrix represents the time-varying trend of the activation level of an activation mode, and the sum of each row represents the activation intensity of the activation mode. The mode with the highest activation intensity is called the primary activation mode, which contributes the most to the force, and the rest are called the secondary activation mode. In this study, the top quarter channels corresponding to the primary activation mode were identified as the optimal channels, and the corresponding sEMG signals were then weighted, averaged and normalized with the maximum-minimum method to generate a one-dimensional sEMG envelope.

#### 2.2.4 Sample segmentation

For the one-dimensional sEMG envelope, a sliding window approach was employed for sample segmentation. A window length of 500 points and a sliding step size of 500 points were utilized. For the force signals, smooth filtering was performed first to remove tiny burrs, then the maximum-minimum method was used for normalization, and finally the same segmentation method was used for sample segmentation.

### 2.3 The establishment of HD-sEMG-force source network

LSTM has proficiency in processing temporal information (Hochreiter and Schmidhuber, 1997). After comparative experiments with a hybrid model of CNN and LSTM, LSTM was used to construct sEMG-force source network in this study due to its lower muscle force estimation. RMSE. The details and results of the comparison are presented in Supplementary Data Sheet 1. The source network model was constructed on the basis of the TensorFlow framework. The samples in the source dataset were randomly divided into a training set, a validation set and a testing set at a ratio of 8:1:1. To determine the structure and parameters of the source network, comparative experiments were conducted on the number of network layers, the number of units in each layer, and the dropout rates. Specifically, we considered two-layer and three-layer LSTM networks, with different combinations of 16–256 units in each layer. For each network structure, dropout values of 0.4, 0.6, and 0.8 were considered. The loss function selected for training the network was the RMSE, which is defined as Formula 2.

$$RMSE=\sqrt{\frac{\sum_{i=1}^{N}{\left(y_{i}-\tilde{y_{i}}\right)}^{2}}{N}}\times 100\%$$ (2)

### 2.4 The establishment of gesture-specific target networks

In practical applications, the choice of transfer learning methods typically depends on the characteristics of both the source and target domains, as well as the task requirements (Pan and Yang, 2010). In the model transfer of neural networks, layer transfer stands out as a widely employed method capable of hastening the training process of new models and enhancing their overall performance. In this study, the target network structure retained the same structure as the source network, with consistent hyperparameter settings. Using data from five target gestures of healthy children, we conducted target network calibration on the well-trained source network via different transfer learning strategies to obtain target networks suitable for the target gestures, namely, gesture-specific target networks.

For the combination of one gesture and one transfer strategy, a leave-one-out cross-validation experiment was conducted for the calibration of the target network. In each iteration, data from 15 HC subjects were chosen as the training and validation sets in 9:1, whereas data from the remaining 1 HC were utilized as the test set, resulting in a total of 16 experimental results. The average RMSE and R^2^ values across these 16 experiments were used to evaluate the performance of the calibrated target network. Goodness-of-fit $R^{2}$ is defined as Formula 3, where x and y are estimated force and measured force, respectively.

$$R^{2}=\frac{\sum_{i=1}^{n}\left(x_{i}-\bar{x}\right)\left(y_{i}-\bar{y}\right)}{\sqrt{\sum_{i=1}^{n}{\left(x_{i}-\bar{x}\right)}^{2}\sum_{i=1}^{n}{\left(y_{i}-\bar{y}\right)}^{2}}}$$ (3)

Furthermore, to investigate the impact of the data volume used for fine-tuning the source network on the performance of the target network, we conducted 7 different training and test ratios on HD-sEMG-force data from 16 HC subjects. The 7 training test ratios were 15:1, 14:2, 12:4, 8:8, 4:12, 2:14, and 1:15, with the specified ratios denoting the proportion of subjects in the training and test sets. No validation set was set.

### 2.5 Application of target networks in hand dysfunction assessment of CP children

Sample data for G1-G5 in CP dataset were separately input into the target networks to obtain force estimation results. The application value of sEMG-based muscle force estimation technology in the hand dysfunction assessment of children with CP was explored by analyzing differences in HD-sEMG-force relationship between CP and HC of the same age, between MACS grades and between upper limb symptom types. In addition, to explore whether sEMG-based muscle force estimation technology can be applied in assessing the effect of rehabilitation training, we analyzed the muscle force estimation results of the tracking experiments on CP6 and CP7.

## 3 Results

### 3.1 Source networks structure and hyperparameters

This study used the Kruskal–Wallis test, a nonparametric alternative to one-way ANOVA, to assess differences between multiple network structures. Following the results, the LSTM network with different numbers of layers achieved good muscle force estimation results under different combinations of dropout and unit numbers, and there was no significant difference in the RMSE (p > 0.05). As shown in Table 2, the model with a three-layer LSTM under a unit combination of 256–128–64 and a dropout of 0.4 achieved the smallest RMSE. Therefore, we ultimately determined that the source network structure comprised 3 layers of LSTM followed by a dense fully connected layer. Batch normalization layer and dropout layer were incorporated after each LSTM layer to expedite convergence and mitigate issues such as gradient explosion or vanishing during training. The dense fully connected layer synthesized features and output the estimation force. The batch size was set to 100, and the initial learning rate was set to 0.001. To prevent overfitting, an early stop technique was implemented. The data from the training set were used to train and obtain the well-trained source network, whose RMSE on the test set was 6.31%.

**TABLE 2:** Force estimation results (RMSE%) for the source network with different numbers of layers, combinations of unit numbers and dropout rates.

Combinations of unit numbers		Dropout		
		0.4	0.6	0.8
LSTM-LSTM	256–128	6.45	6.61	6.46
	128–64	6.47	6.46	6.72
	64–32	6.43	6.55	7.61
LSTM-LSTM-LSTM	256–128–64	6.31 ★	6.34	6.45
	128–64–32	6.38	6.46	6.73
	64–32–16	6.44	6.70	9.87

### 3.2 Performance testing of target networks based on different transfer learning strategies

#### 3.2.1 Selection of transfer learning strategy

**FIGURE 3:**
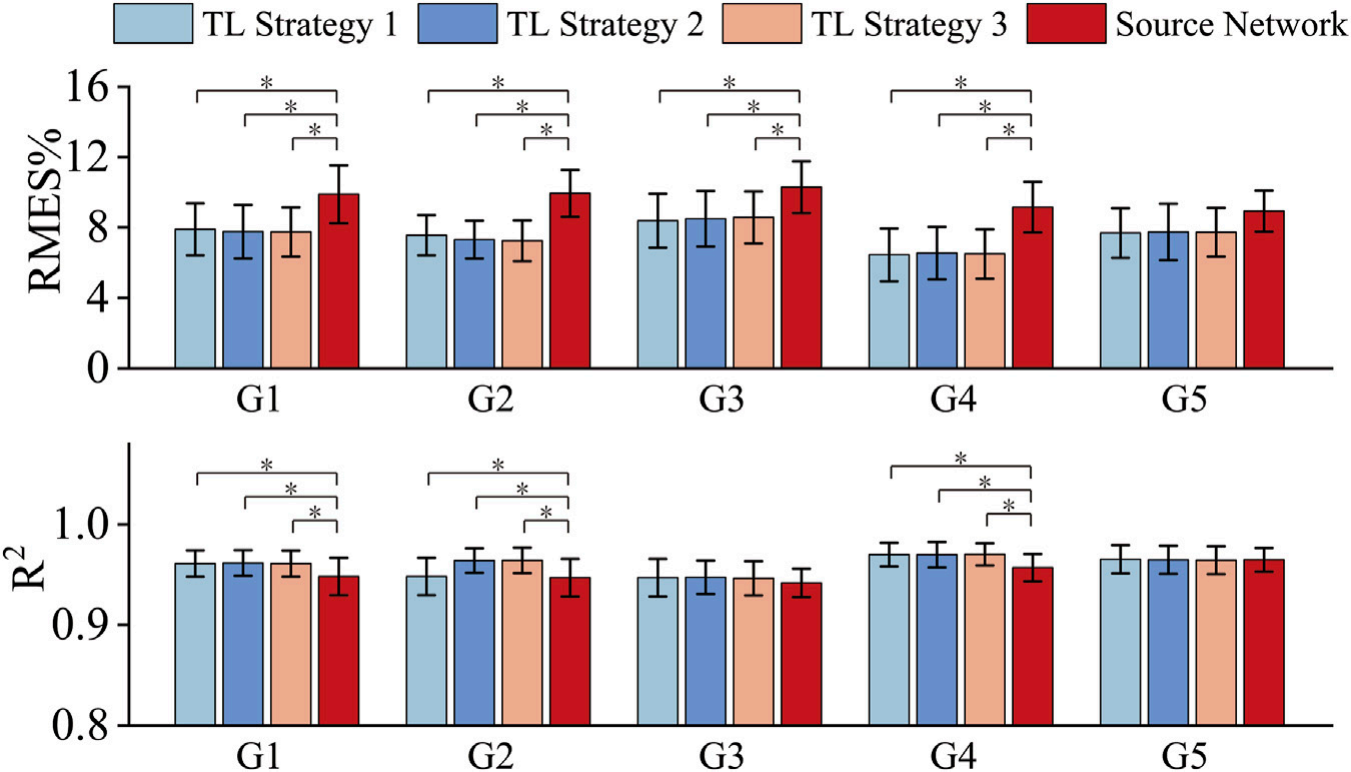
RMSE and R^2^ obtained by using three transfer strategies and the source network. * represents a significance level of <0.016.

Based on the determined structure of the source network, the following three transfer strategies (TL strategy 1–3) were adopted. TL strategy 1: Transfer all layer parameters with all layers trainable; TL strategy 2: Transfer all layer parameters while freezing the first layer; TL strategy 3: Transfer all layer parameters while freezing the first two layers. Directly using the well-trained source network (labeled ‘Source Network’) was used as a comparison method. Figure 3 shows the experimental results for the five target gestures under the three transfer learning strategies and using source network. The results show that fine-tuning the layer parameters through a transfer learning strategy (TL strategy 1–3) achieved better muscle force estimation accuracy than directly using the well-trained source network (Source Network). For the five target gestures, the mean RMSE values of the target networks obtained using TL strategy 1-3 were 2.09%, 2.57%, 1.81%, 2.66% and 1.21% lower than using source network, respectively. These results indicate that the idea of using transfer learning and HC data to fine-tune the source network to establish gesture-specific target networks was effective. Additionally, for G1-G5, significant analysis was conducted on TL strategies 1-3 and Source Network based on the results of RMSE and $R^{2}$ , respectively. Based both on RMSE and $R^{2}$ , repeated-measures ANOVA with the Friedman test indicated no significant differences between TL strategies 1–3 (P > 0.05). Based on RMSE, for G1-G4, all TL strategies showed significant differences when compared to the source network (P < 0.016, according to Bonferroni corrected significance level of multiple comparisons). For G5, there was no significant difference between TL strategies and the source network (p > 0.016). Based on $R^{2}$ , for G1, G2 and G4, all TL strategies showed significant differences when compared to the source network (P < 0.016), for G3 and G5, there was no significant difference between TL strategies and the source network (p > 0.016). Upon comparison, we determined the optimal transfer strategies for each gesture as follows: For G1 and G2, TL strategy 3 was selected. For G3, G4, and G5, TL strategy 1 was selected.

#### 3.2.2 Validation of transfer learning strategy effectiveness

**FIGURE 4:**
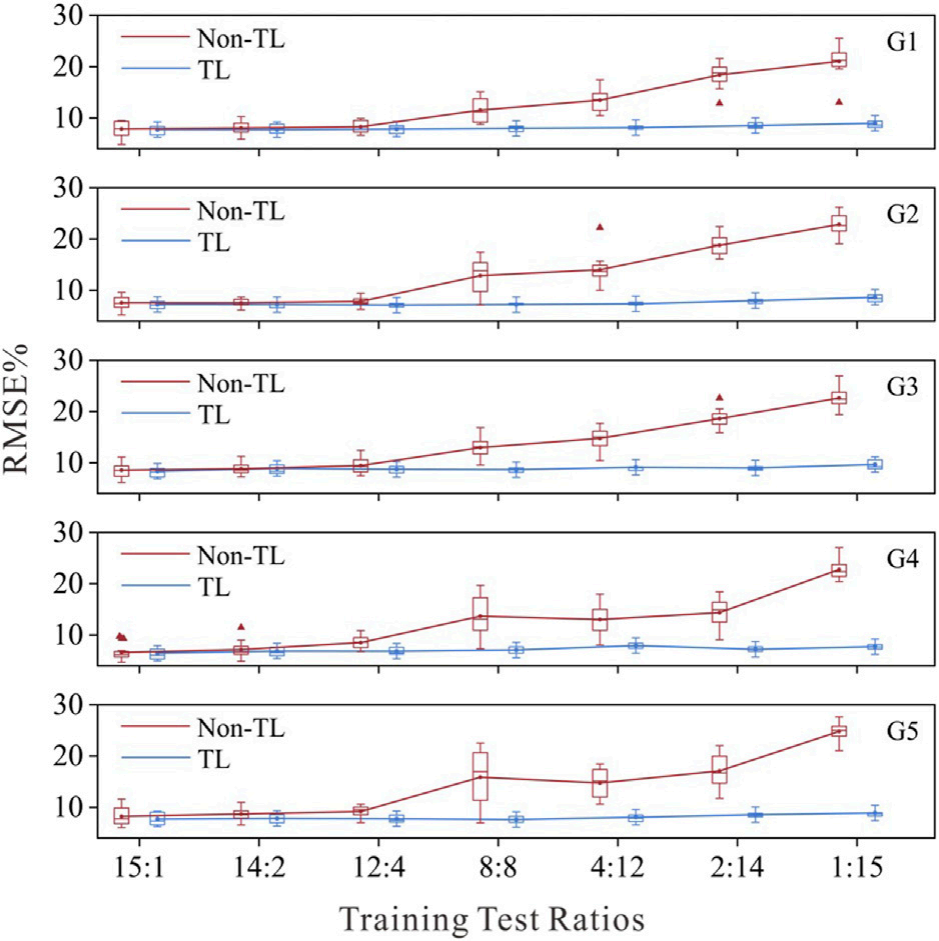
Force estimation results with the TL strategy and non-TL strategy under different training test ratios.

To mitigate the impact of random sampling on the results, we employed a 16-fold cross-validation method for each training test ratio, and the estimation results of the model were averaged across the 16 iterations. For comparison, the muscle force estimation results of the five target gestures in each training test ratio are illustrated in Figure 4. TL refers to TL strategy 3 for G1 and G2 and TL strategy 1 for G3, G4 and G5. Non-TL refers to using only the same structure as the source network but not migrating the parameters of the well-trained source network.

Figure 4 shows that as the number of training samples decreases, the performance of the target network using the Non-TL strategy deteriorates, whereas the muscle force estimation errors of each target network using the TL strategy remain relatively low. The RMSE for all target networks remained below 10%, even when they were calibrated with only data from one subject. This performance, which could achieve superior performance with only a small amount of data calibration, is highly important for clinical practice. The average test error of the five gesture-specific target networks obtained when the data of the 15th subject are used for transfer learning is the smallest, average RMSE = 8.07 ± 0.30%. Therefore, following the optimal principle, sEMG-force data from Subject 15 were ultimately used to train and obtain five gesture-specific target networks named M1-M5. On the M1-M5 model, the average muscle force estimation RMSE of the remain 15 subjects were as follows: RMSE = 7.99 ± 2.74 for M1; RMSE = 7.78 ± 3.09 for M2; RMSE = 9.19 ± 2.90 for M3; RMSE = 7.03 ± 2.81 for M4; RMSE = 8.36 ± 3.49 for M5.

### 3.3 Analysis of the relationship between HD-sEMG and muscle force in children with CP based on target networks

#### 3.3.1 Comparison between HC and CP

**FIGURE 5:**
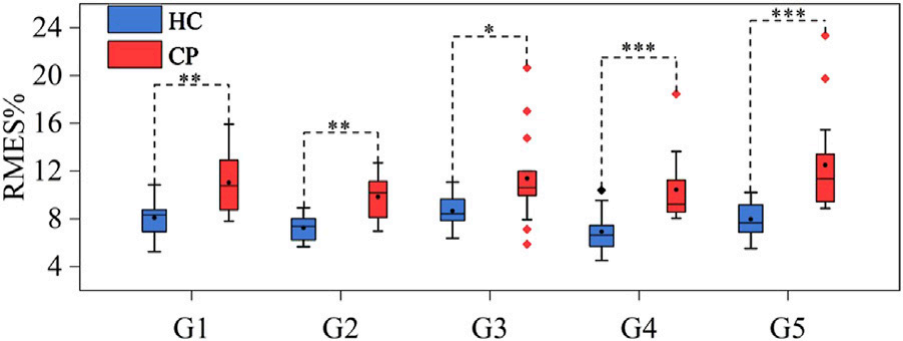
Muscle force estimation results for G1-G5 in the HC and CP groups. *** represents a significance level of <0.001, ** represents a significance level of <0.01, and * represents a significance level of <0.05.

A significance analysis was conducted on the age of HC and CP groups using independent sample t-test, and there was no significant age difference between the two groups (p > 0.05). To explore whether there are differences in HD-sEMG‒force relationship between CP and HC groups, data from CP and HC groups (HC15 was excluded) were input into M1-M5 respectively to obtain the force estimation RMSE. For each gesture, the average RMSE value of each subject’s eight repetitions were recorded, and the average RMSE of all the subjects in each group was calculated as the RMSE of that group. Mann-Whitney U test was used to conduct a significance analysis of pairwise comparisons between CP group and HC group. Figure 5 shows the muscle force estimation results of five target gestures in the form of box plots. The results reveal significant differences (p < 0.05 for G3, p < 0.01 for G1-G2 and p < 0.001 for G4-G5) in all five gestures between the CP and HC groups. Compared with the HC group, the CP group achieved a higher RMSE with a larger fluctuation range. Specifically, for G1, the ranges of RMSE in HC and CP groups were 5.26%–10.86% and 7.80%–15.91%, respectively, with CP group being 2.84% higher than HC group; for G2, the ranges of RMSE in HC and CP groups were 5.66%–8.93% and 6.97%–12.68%, respectively, with CP group being 2.53% higher than HC group; for G3, the ranges of RMSE in HC and CP groups were 6.36%–11.09% and 5.87%–20.63%, respectively, with CP group being 2.64% higher than HC group; for G4, the ranges of RMSE in HC and CP groups were 4.50%–10.39% and 8.05%–18.45%, respectively, with CP group being 3.5% higher than HC group; and for G5, the ranges of RMSE in HC and CP groups were 5.51%–10.22% and 8.86%–23.33% respectively, with CP group being 4.4% higher than HC group. In addition, after repeated-measures ANOVA with the Friedman test, there was no significant difference (p > 0.05) in RMSE of different gestures in both HC and CP group.

#### 3.3.2 Comparison of different MACS grades

**FIGURE 6:**
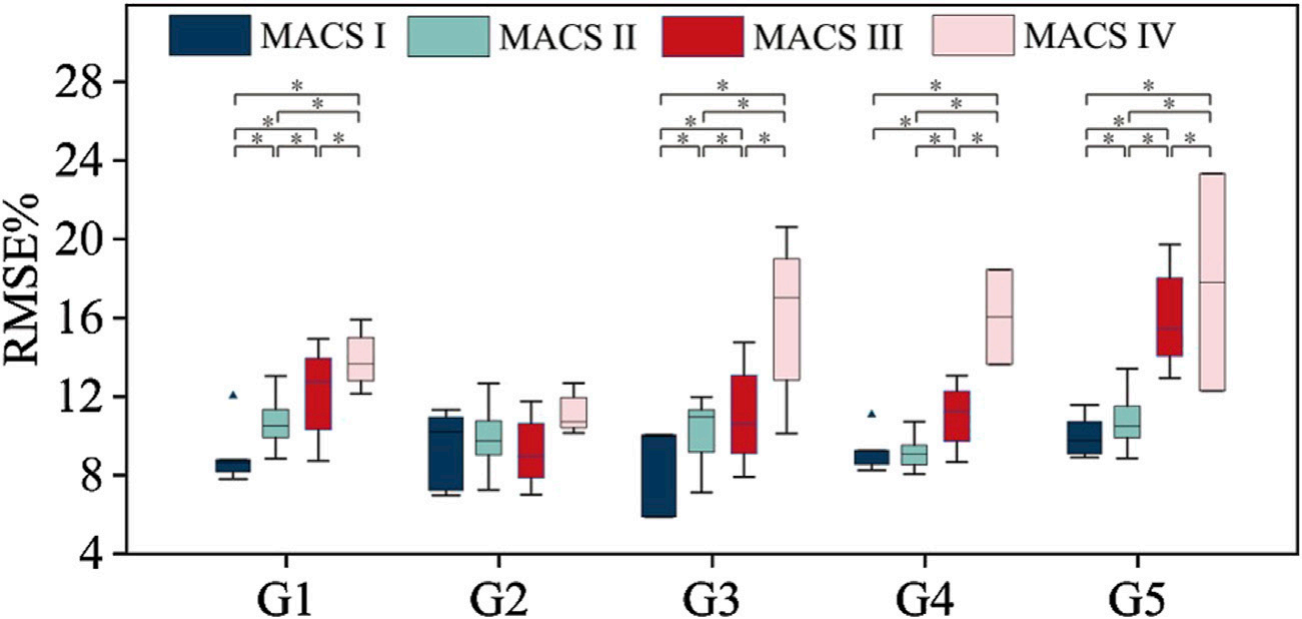
Muscle force estimation results according to MACS grading. * represents a significance level of <0.0083.

To explore whether the abnormal HD-sEMG-force relationship can be used to assess the degree of motor impairment, we compared the muscle force estimation results of CP subjects with different MACS grades. As shown in Table 1, MACS grades 1, 2, 3, and 4 had 5, 5, 3, and 3 subjects, respectively. Figure 6 shows the muscle force estimation RMSE of the five target gestures according to the MACS grading. For each grade, the results presented were the means and standard deviations of all the subjects.

For gestures G1, G3, and G5, the muscle force estimation RMSE values were significantly influenced by MACS level. The Kruskal–Wallis test result was p < 0.05. Dunn’s test was used for further pairwise testing, and Bonferroni correction was used to adjust the significance level for multiple comparisons. The Dunn’s test results between each two MACS grades were p < 0.0083. For gesture G4, The Kruskal–Wallis test result was p < 0.05, in the Dunn’s test, expect for no significant difference between MACS Ⅰ and MACS Ⅱ grades (p > 0.0083), there were significant differences between every other two grades (p < 0.0083). In summary, higher MACS levels had higher average RMSE or standard deviations. For G1, G3, G4, and G5, the mean RMSE of MACS Ⅰ group was in the range of 8.63%–10.01%, that of MACS Ⅱ group was 9.03%–10.71%, that of MACS Ⅲ group was 11.00%–16.05%, and that of MACS Ⅳ group was 13.90%–17.81%. However, for G2, no significant difference (The Kruskal–Wallis test result was p > 0.05) was observed between MACS grades. This discrepancy could be attributed to the fact that G2 required less fine motor function than the other gestures do. Consequently, CP subjects across different MACS grades demonstrated relatively stable neuromuscular control ability for G2.

#### 3.3.3 Comparison of different upper limb symptoms

**FIGURE 7:**
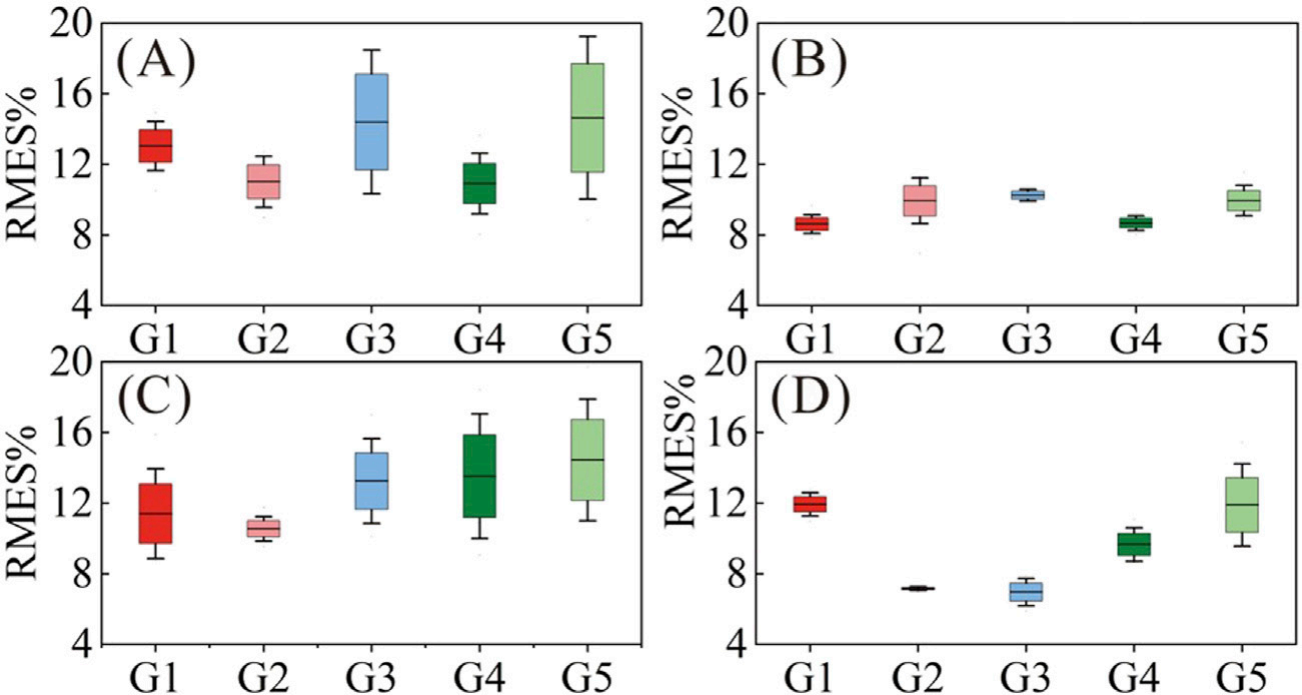
Muscle force estimation results according to upper limb symptoms. **(A)** Type I; **(B)** Type II; **(C)** Type III; **(D)** Type IV.

To explore whether the abnormal HD-sEMG-force relationship is related to upper limb symptoms, we compared the muscle force estimation results of CP subjects with different upper limb symptoms. Figure 7 shows the muscle force estimation results of the five target gestures according to upper limb symptoms. As shown in Table 1, Type I, Type II, Type III and Type IV had 4, 5, 4, and 3 subjects, respectively. The results were presented as the means and standard deviations of the subjects of each type.

**FIGURE 8:**
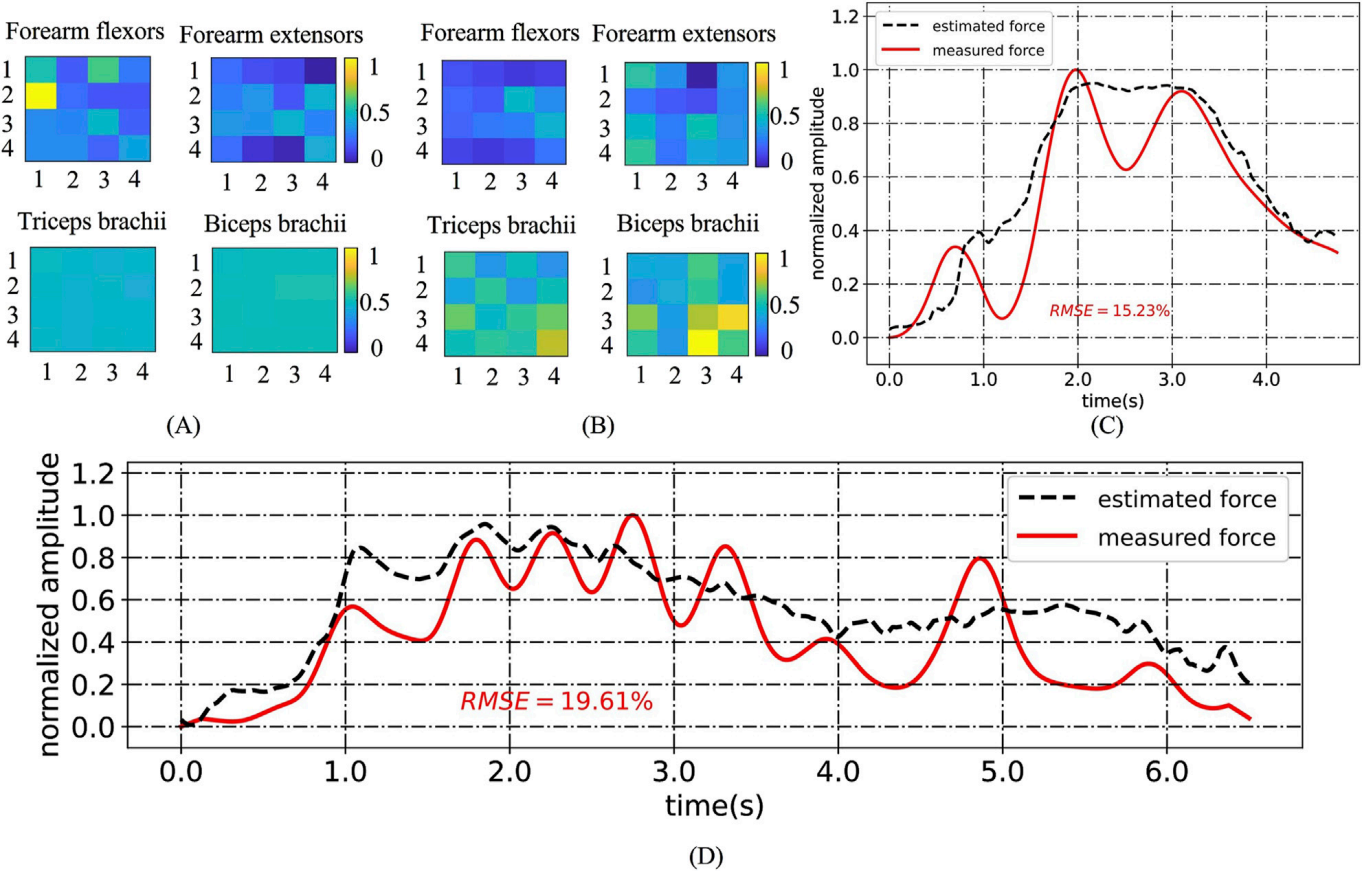
**(A)** HD-sEMG heatmaps for G1 of HC2; **(B)** HD-sEMG heatmaps for G1 of CP1; **(C)** An example of an “M”-shaped tremor; **(D)** Muscle force estimation example for CP14 under G3.

Type I (CP1, CP6, CP11 and CP14) had relatively high RMSE (10.92%–14.64% on average) with large standard deviations (1.61%–5.32%) for all gestures, especially for G1, G3 and G5. We believe that this was related to upper limb symptoms characterized by muscle stiffness and abnormal muscle tone. Taking CP1 as an example, Figures 8A, B compares its HD-sEMG heatmap to that of HC2. CP1 revealed abnormal activation in the upper arm muscles (biceps and triceps). The muscle activation pattern of HC2 was predominantly distributed in the forearm flexor muscles, indicating their pivotal role in executing this gesture. In contrast, the muscle activation pattern of CP1 was primarily concentrated in the upper arm area, which might lead to a misalignment between its HD-sEMG and muscle force. Additionally, within this type, CP14 presented with dyskinetic cerebral palsy. During the execution of high-intensity gestures such as G3 and G5, CP14 manifested significant involuntary movements, resulting in pronounced fluctuations in force output. As illustrated in Figure 8D, the muscle force curve rapidly changes, thereby diminishing the tracking performance of the muscle force estimation mode.

Type Ⅱ subjects exhibited superior hand motor function compared to the other three types. As shown in Figure 7, Type II (CP2, CP3, CP8, CP15 and CP16) had relatively low RMSE (8.62%–10.26% on average) for all gestures, with small standard deviations (0.36%–1.54%), which were closest to the level of healthy children. However, owing to inadequate control over force, Type II subjects often experienced “M”-shaped tremors (as shown in Figure 8C) when performing gestures, posing a challenge for accurately tracking force output. Accordingly, CP2, CP8, and CP15 displayed elevated RMSE, specifically in G2.

For Type III (CP4, CP5, CP12, and CP13), except for G2 (average RMSE of 10.55%), the other four gestures had relatively high RMSE (11.41%–14.45%) and large standard deviations (2.76%–3.84%), largely attributed to symptoms such as wrist drooping and finger interlocking. For G1, G3, G4 and G5, the inward clasp of the fingers caused the subject’s thumb to continue bending, hindering the complete exerting force process. The suspension of the wrist hindered the subject from fully grasping and applying force, necessitating compensatory activation of the entire upper limb muscles. The stiffness of the wrist required the help of the upper arm to enable the subject to achieve a grasping posture. For G2, the involvement of the other four fingers mitigated the impact of the thumb inward clasp, resulting in slightly improved force estimation results.

Type IV subjects (CP7, CP9, and CP10) exhibited poor ulnar radial separation, characterized by ulnar lateral movement accompanied by radial hand movements. As shown in Figure 7, Type IV had (6.97%–11.94%) RMSE with small standard deviations (0.11%–2.53%), and G2 and G3 were significantly lower than those of the other three gestures. During the execution of G1 and G5, simultaneous bending of the other three fingers occurs, which in turn leads to an increase in the force estimation error. However, the impact on G2 and G3 was relatively limited.

#### 3.3.4 Comparison before and after rehabilitation training

**FIGURE 9:**
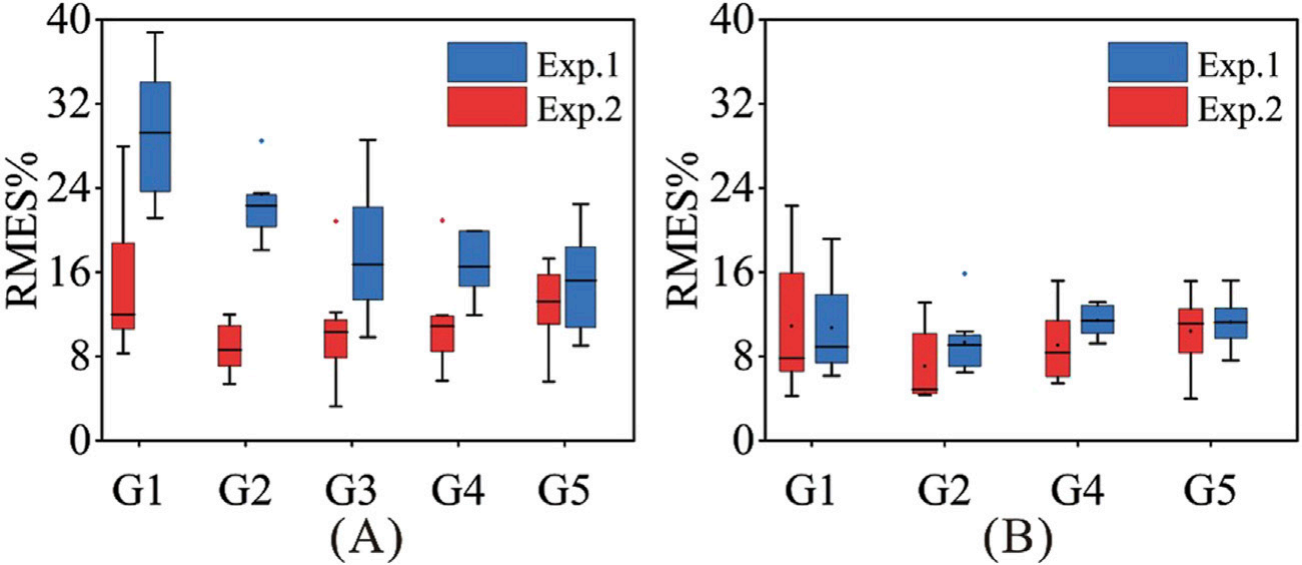
The force estimation results of Exp.1 and Exp.2. **(A)** CP6; **(B)** CP7.

The results of the tracking experiments on CP6 and CP7 are shown in Figure 9. The RMSE of force estimation for CP6 was relatively large (15.02%–29.24%) in Exp. 1 but decreased by approximately 1.88%–14.37% in Exp. 2. The reason is that at Exp. 1, CP6 had just started rehabilitation training for approximately 4 months and had severe hand dysfunction and poor gesture completion. After 6 months of continuous and effective rehabilitation training, the symptoms of hand dysfunction in CP6 patients were alleviated, and abnormal hand posture was reduced. Therefore, good force estimation results were obtained in Exp. 2. In contrast, CP7 received long-term scientific rehabilitation training after birth, and his hand movement function was in a stable state during Exp. 1 and Exp. 2, so the force estimation results of the two experiments did not change much.

## 4 Discussion

With the goal of applying sEMG-based muscle force estimation technology to assess motor dysfunction in CP children in the clinic, this work consists of three main parts, namely,: establishing a HD-sEMG-force source network, constructing HD-sEMG-force target networks for five rehabilitation gestures, and assessing the hand dysfunction of children with CP. In this section, we discuss the progressiveness, theoretical value and clinical application value of the research results obtained in this study.

Firstly, this study established a HD-sEMG-force source network using three adult datasets covering different force modes and different subjects, as well as an LSTM model. As mentioned in the introduction, sEMG-based muscle force estimation technology is currently a research hotspot in the field of biomedical engineering. Various effective sEMG-based muscle force estimation methods have been proposed, and their performances were usually tested in subject-related way or subject-independent way depending on whether the training and testing data come from the same subjects. Table 3 summarizes recent researches in this field, where “No.Dataset” represents the number of datasets used, “Y” and “N” represent respectively the work was carried out in subject-independent way or in subject-related way. Under subject-related way, Wahid et al. (2024) employed a combined CNN and LSTM model to achieve a RMSE = 2.3% in the triangular force mode task (Wahid et al., 2024). Similarly, Jiang et al. (2023) developed a force estimation model using deep forests for the triangular mode force task (Jiang et al., 2023). Notably, they utilized two datasets collected from the same subjects on different days, with one dataset used for training and the other for testing, resulting in a RMSE of 8.0% ± 2.3%. Our model achieved RMSE = 6.31% under subject-related way. In comparison to Wahid’s work, our model has the advantage of being validated across multiple force mode tasks using data from different datasets and more subjects, demonstrating superior generalization capability. Compared with Jiang’s model, our network is applicable to a wider range of force modes and offers higher accuracy. Under subject-independent way, Hajian et al. (2021) presented the “CNN-FLF” model, which is a CNN model with feature level fusion (Hajian et al., 2021). In the rectangular force mode task, their model achieved an accuracy of NMSE = 1.60 ± 3.69%, corresponding to a RMSE of 12.6% ± 19.21%. Xu et al. (2018) utilized a CNN-LSTM hybrid model, referred to as “C-LSTM,” to estimate force in the “increasing-plateau” force mode task, achieving RMSE = 8.67% (Xu et al., 2018). In contrast, our model achieved RMSE = 9.64 ± 1.47% under subject-independent way, which is better than Hajian’s results but slightly lower than those of Xu’s. One of the reasons is that our model was trained on adult data, but the test results obtained on children’s data, leading to increased error due to differences in subject population. In addition, most of these previous works focused on single force mode, only Simon et al. (2024) used time-varying force mode, but unfortunately their accuracy was not satisfactory (Simon et al., 2024). In general, the strength of our model lies in its applicability to multiple force modes data from three datasets, enhancing its generalization. Meanwhile, the accuracy of our model is considered satisfactory.

**TABLE 3:** Summary of Recent Research on sEMG-Based Force Estimation** “C-LSTM” is a combination model of Convolutional Neural Network (CNN) and LSTM, “CNN-FLF” is a CNN model with feature level fusion, and “TCN” is an abbreviation for Temporal Convolutional Network.

Year	Author	Subject	Model	Force mode	No.Dataset	Subject-independent	Accuracy
2018	Xu et al.	8M	C-LSTM	increasing-plateau	1	Y	RMSE = 8.67 ± 1.14%
2021	Hajian et al.	5F,8M	CNN-FLF	rectangle	1	Y	NMSE = 1.60 ± 3.69%
2023	Jiang et al.	8F,12M	Deep Forests	triangle	2	N	RMSE = 8.0 ± 2.3%
2024	Simon et al.	50	TCN	time-varying	1	Y	RMSE = 29.9 ± 13.1%
2024	Wahid et al.	5M	CNN-LSTM	triangle	1	N	RMSE = 2.3%
Our work		3F,29M	LSTM	increasing-plateau, sine, rectangle, random	3	N	RMSE = 6.31%
						Y	RMSE = 9.64 ± 1.47%

Secondly, this study established five gesture-specific target networks by calibrating the parameters of the well-trained source network using transfer learning technique and the sEMG-force data of five target gestures from 16 healthy children. Compared with directly using the well-trained source network, the gesture-specific target networks reduced the RMSE of force estimation of the five target gestures by 2.14%, 2.70%, 1.91%, 2.71%, and 1.24%, confirming the effectiveness of using the transfer learning technique to improve muscle force estimation accuracy. Compared with directly using gesture data from healthy children for force estimation network training, the advantage of using a transfer learning technique is that it only requires a small amount of data to obtain satisfactory force estimation accuracy. When transfer learning strategies were adopted, an average RMSE of less than 10% was obtained when only one healthy child’s data were used as the training set. However, when only healthy children’s data were used to train the source network structure, the performance of the force estimation model was closely related to the training test ratio. Relatively satisfactory performance could only be achieved when the training test ratio was 12:4. Clinical applications have high requirements for the accuracy of muscle estimation models, however, there are significant difficulties in collecting a large amount of healthy subject data for model training. The model calibration scheme based on the transfer learning strategy proposed in this study greatly reduces the training data volume requirement, providing a feasible solution for promoting the application of this technology. Hajian et al. also attempted to extend the force estimation model to new users using transfer learning techniques (Hajian et al., 2024). Their research results revealed that under isotonic, isokinetic, and dynamic conditions, compared with the leave-one-subject-out (LOSO) case, the transfer learning technique increased the $R^{2}$ by 60.81%, 190.53%, and 199.79%, respectively. Compared with the intra subject case, the transfer learning technique increased $R^{2}$ by 13.4%, 36.88%, and 45.51%, respectively. Their research also confirms the advantages of using transfer learning to alleviate the training burden on new users.

Finally, this paper explored the feasibility of applying the proposed HD-sEMG-based muscle force estimation scheme to CP hand dysfunction assessment. The research results first confirmed that the target networks can be used for rough assessment of the force level during gesture execution in CP children in clinical practice, with an error of less than 20%. Moreover, the CP group was confirmed to have an abnormal HD-sEMG-force relationship compared to that of the same-age HC group, providing a basis for assessing the degree of CP hand dysfunction. By comparing the muscle force estimation results of CP subjects with different MACS grades, we found that the muscle force estimation error was significantly affected by the MACS grading. In summary, the higher the MACS grade is, the higher the average RMSE or standard deviation. However, not all gestures have a good correlation between the degree of deviation of the HD-sEMG-force relationship and MACS grading. When designing target gestures for assessing hand dysfunction, it is necessary to avoid gestures such as G2, which do not involve fine motor skills. Moreover, the results demonstrated that muscle stiffness and abnormal muscle tone, “M”-shaped tremors, wrist drooping and finger interlocking, and poor ulnar radial separation can cause abnormal HD-sEMG-force relationship in CP children. Therefore, the degree to which the HD-sEMG-force relationship deviates from that of the healthy population can be used to assess these clinical symptoms. To achieve this, it is also necessary to select suitable gestures. For example, when symptoms of ulnar radial nonseparation are assessed, gestures that are less affected by this symptom, such as G2 and G3, should be avoided; when symptoms of wrist drooping and finger interlocking are assessed, G2 should be avoided. Finally, the proposed scheme can also be used to track and assess the rehabilitation process of CP patients, and the target networks can be used to assess the stability of hand function in children with CP. The results of CP7 indicated that once the patient establishes their own force generation mode, the effect of rehabilitation training decreases.

## 5 Conclusion

The main contribution of this study is to propose a novel HD-sEMG-force estimation framework and explore the feasibility of applying the proposed framework to the assessment of motor dysfunction in CP children in the clinic. Specifically, a high-precision source network model with high generalizability was established. On this basis, gesture-specific target networks were established using transfer learning techniques and data from healthy children, and their clinical value for assessing hand dysfunction in CP children was verified. This study has laid a foundation for promoting the application of sEMG-based muscle force estimation technology in clinical practice and can provide a quantifiable means for assessing motor dysfunction. The primary limitation of this study lies in the labor-intensive preprocessing of sEMG signals, which impedes its feasibility for real-time application in clinical settings. Furthermore, the need to collect sEMG-force data from healthy children to fine-tune the source network presents additional challenges for clinical implementation. Future research will focus on the development of advanced neural networks capable of extracting both spatial and temporal features to replace existing channel optimization algorithms. Additionally, expanding the training to include a broader range of datasets can enhance the model’s generalizability, potentially eliminating the need for fine-tuning the source network.

## Data Availability

The raw data supporting the conclusions of this article will be made available by the authors, without undue reservation.
