# Supplementary material for: Feasibility study on the application of HD-sEMG-based force estimation technology in the assessment of hand dysfunction in cerebral palsy
Source: Front Bioeng Biotechnol. 2025 Apr 2;13:1580098. doi: 10.3389/fbioe.2025.1580098 (PMC11999938; doi:10.3389/fbioe.2025.1580098)
Supplement: Supplementary file 1 [file DataSheet1.docx]

Supplementary Material

# Supplementary Information on determining the source network structure

In this paper, we considered two representative candidate network structures, namely LSTM structure and CNN-LSTM structure, when constructing the source network model based on the TensorFlow framework. The LSTM structure consists of N layers of LSTM and a Dense fully connected layer. At the same time, after each layer of LSTM, Batch normalization layer and dropout layer were incorporated to expedite convergence and mitigate issues such as gradient explosion or vanishing during training. The dense fully connected layer synthesized features and output the estimation force. The CNN-LSTM structure consists of N layers of CNN, M layers of LSTM, and one layer of Dense. Batch Normalization layer and Dropout layer are also added after the inner CNN layer and LSTM layer. The schematic diagram of two source network structures is shown in Supplementary Figure 1.

The samples in the source dataset were randomly divided into a training set, a validation set and a testing set at a ratio of 8:1:1. Firstly, the impact of different layers of network structures on network accuracy was compared. Three layers were considered for LSTM structure and CNN-LSTM structure, respectively, as shown in the supplementary Table I. For each network structure, dropout values of 0.4, 0.6, and 0.8 were considered. The estimation results on the testing set are shown in supplementary Table II. From the results, it can be concluded that the mean force estimation RMSE of two-layer LSTM and three-layer LSTM are slightly lower than those of other network structures. Therefore, further experiments were conducted on the source networks of two-layer LSTM and three-layer LSTM structures to determine the optimal combination of unit numbers. This section is presented in detail in the main body of the paper, and the final determined network structure and number of units are shown in Supplementary Figure 2.

# Supplementary Figures and Tables

## Supplementary Figures


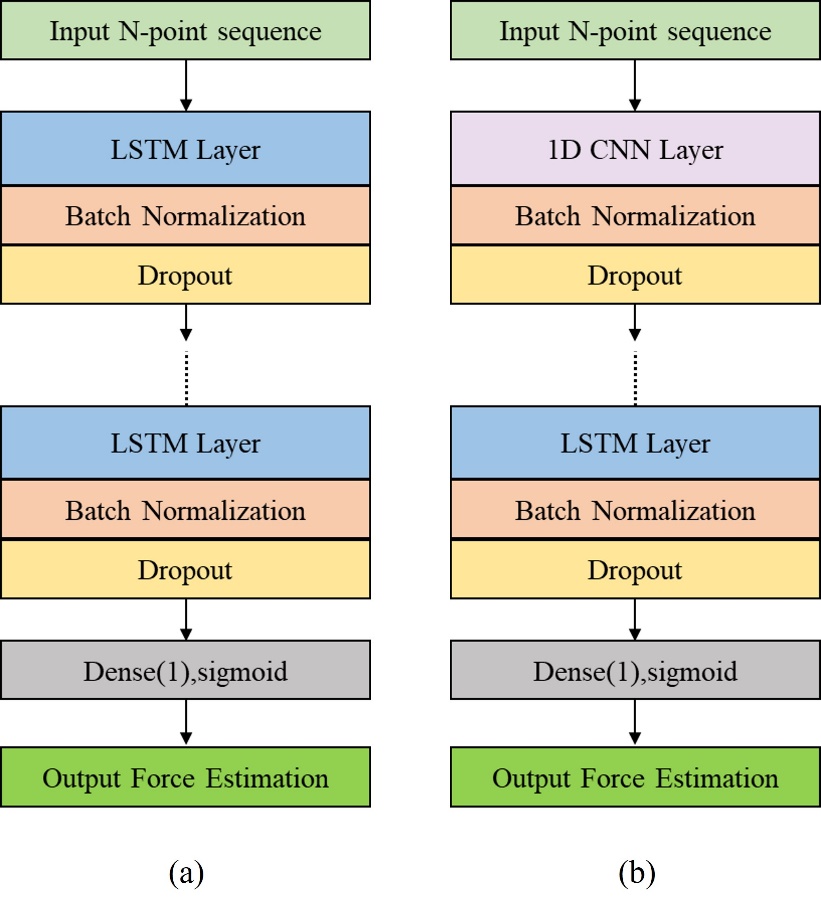


**Supplementary Figure 1.** (a) The schematic of LSTM structure. (b) The schematic of CNN-LSTM structure.


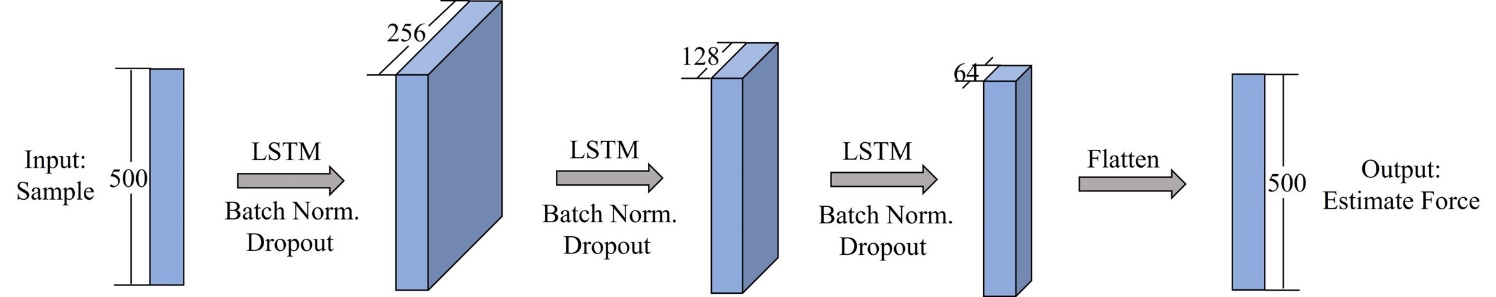


**Supplementary Figure 2.** Schematic diagram of the source network structure.

## Supplementary Tables

Table I The number of units or kernels per layer in the structure of LSTM and CNN-LSTM.

| **LSTM structure** | | **CNN-LSTM structure** | |
| --- | --- | --- | --- |
| **layer** | **unit** | **layer** | **unit/kernel** |
| LSTM | 128 | CNN-LSTM | 32-128 |
| LSTM-LSTM | 128-64 | CNN-CNN-LSTM | 32-64-128 |
| LSTM-LSTM-LSTM | 128-64-32 | CNN-LSTM-LSTM | 32-128-64 |

Table II RMSE (%) of force estimation for source networks with different network structures and Dropouts.

| **Network structure** | **Dropout** | | |
| --- | --- | --- | --- |
|  | 0.4 | 0.6 | 0.8 |
| LSTM | 8.362 | 7.330 | 7.257 |
| LSTM-LSTM | 6.474 | 6.455 | 6.718 |
| LSTM-LSTM-LSTM | 6.379 | 6.456 | 6.728 |
| CNN-LSTM | 6.435 | 6.455 | 7.158 |
| CNN-CNN-LSTM | 6.809 | 6.643 | 7.711 |
| CNN-LSTM-LSTM | 6.445 | 7.141 | 9.449 |
